# Artificial intelligence in oncology drug development and management: a precision medicine perspective

**DOI:** 10.3389/fonc.2025.1609827

**Published:** 2025-12-04

**Authors:** Caixia Fang, Pengfa Zhou, Xuerong Zhang, Yongsheng He, Qingwei Yang

**Affiliations:** 1 Pharmacy Clinical Research Centre, Qingyang People’s Hospital, Qingyang, China; 2 Department of Pharmacy, Qingyang People’s Hospital, Qingyang, China; 3 Department of Medical Oncology, Qingyang People’s Hospital, Qingyang, China

**Keywords:** artificial intelligence, oncology, precision medicine, drug development, clinical trials, toxicity prediction, pharmacovigilance, real-world evidence

## Abstract

The management of oncology drugs is inherently complex, facing challenges such as high development costs, prolonged timelines, and substantial inter-patient heterogeneity. Recent advances in artificial intelligence (AI) have introduced transformative capabilities across the entire cancer drug lifecycle—from target discovery and compound screening to clinical trial optimization, individualized therapy, toxicity management, supply chain logistics, and regulatory oversight. AI enables precise target identification, accelerates virtual drug screening and molecular design, and enhances clinical trial efficiency through intelligent patient stratification and adaptive protocols. Moreover, AI facilitates personalized treatment decision-making, early prediction of drug resistance, and real-time toxicity surveillance, while improving pharmacovigilance and post-market drug evaluation using real-world data. Here, “post-market drug evaluation” refers to real-world effectiveness and safety assessment using spontaneous reports (e.g., FAERS/VigiBase) and EHR/claims-based outcomes, rather than cost-effectiveness analyses. Examples include EHR-NLP to surface immune-related adverse events, AI-assisted trial recruitment and adaptive designs, and individualized dosing frameworks (e.g., CURATE.AI). Despite its enormous promise, AI-driven oncology drug management faces notable challenges in data integration, model interpretability, clinical translation, fairness, and regulatory governance. This review comprehensively summarizes the current applications of AI in oncology pharmacology, highlights key opportunities and barriers, and explores future directions at the intersection of AI, precision medicine, and cancer therapeutics. Future priorities include prospective multi-site evaluations, fairness auditing, and continuous post-market algorithmovigilance.

## 1 Introduction

The development and management of oncology drugs is an extraordinarily complex, costly, and time-consuming process. It typically takes 10–15 years and over one billion dollars to bring a novel compound from candidate identification to market approval (1). This review focuses on AI in anti-cancer drug development and clinical management, spanning discovery, clinical translation, and post-marketing safety/effectiveness, rather than AI in general cancer pathobiology. Despite such substantial investments, the failure rate in oncology drug development remains alarmingly high—studies have reported attrition rates exceeding 95% during clinical development (2). This leads to an annual waste of $50–60 billion on failed oncology clinical trials, in which numerous patients participate without yielding effective therapies (2–4). The inherent heterogeneity and inter-patient variability in tumors further complicate this landscape: genetic and phenotypic discrepancies exist not only across different patients but also among tumor cells within the same individual (3, 5). This diversity often renders standardized treatment regimens ineffective, limiting therapeutic efficacy while increasing the likelihood of adverse effects (5–7). Consequently, oncology drug development is hindered by high costs, prolonged timelines, and profound individual variability.

Against this backdrop, AI has emerged as a transformative tool garnering increasing attention in biomedicine (8). The accumulation of vast, multidimensional datasets—from genomics and proteomics to electronic health records and scientific literature—presents a unique opportunity for AI to extract actionable insights through advanced data processing and pattern recognition capabilities (9–11). By leveraging complex algorithms to mine large-scale data, AI can enhance efficiency, reduce development costs, and improve success rates throughout the drug development lifecycle (12–14). For instance, AI enables the identification of novel therapeutic targets, optimization of molecular design, and personalization of treatment strategies based on patient-specific features. This data-driven paradigm is gradually supplanting traditional empirical approaches, offering faster and more precise development pipelines (12, 15, 16). In the context of highly heterogeneous malignancies, AI provides indispensable tools to advance individualized therapy.

Despite its tremendous promise, multiple challenges persist in oncology drug management. One critical obstacle is drug resistance. Tumors are highly adaptive and often acquire resistance shortly after initial treatment efficacy, leading to disease recurrence. In fact, therapeutic resistance is widely regarded as the greatest barrier in cancer treatment (17–19). Clinicians frequently encounter patients who exhibit poor responses to multiple therapies, resulting in treatment failure and poor prognosis (20, 21). Predicting and overcoming resistance is therefore a central challenge in oncology. Another major concern is the prediction of drug toxicity. Clinical trials cannot fully exclude unforeseen serious adverse reactions due to limited exposure to diverse populations. Effective toxicity prediction is essential for safeguarding patient safety, yet traditional methods struggle to accurately identify high-risk signals amid complex molecular and clinical data (22). In summary, drug resistance and toxicity significantly undermine treatment efficacy and safety in cancer therapy, necessitating novel solutions—such as AI—to address these urgent issues. Unlike prior reviews focused on isolated stages, we synthesize end-to-end applications and explicitly integrate post-marketing pharmacovigilance/RWE with upstream discovery across the lifecycle.To avoid ambiguity between “drugs to study cancer” and “drugs against cancer,” our focus is on AI for anti-cancer drug development and clinical use across the lifecycle (target discovery, screening/design, clinical trials, precision therapy/dosing, toxicity/safety, supply chain, and post-marketing pharmacovigilance/RWE).

## 2 Method

We conducted a narrative review using PubMed, Scopus, and Web of Science covering January 2020–April 2025. Inclusion criteria: oncology-focused primary or methodological studies applying AI to drug discovery/development, clinical trials, precision therapy, toxicity/safety, supply chain, or pharmacovigilance. Exclusion: non-oncology scope or opinion-only pieces without methods. Two authors independently screened titles/abstracts and full texts; disagreements were resolved by consensus.

## 3 Applications of AI in oncology drug development

**Figure 1:**
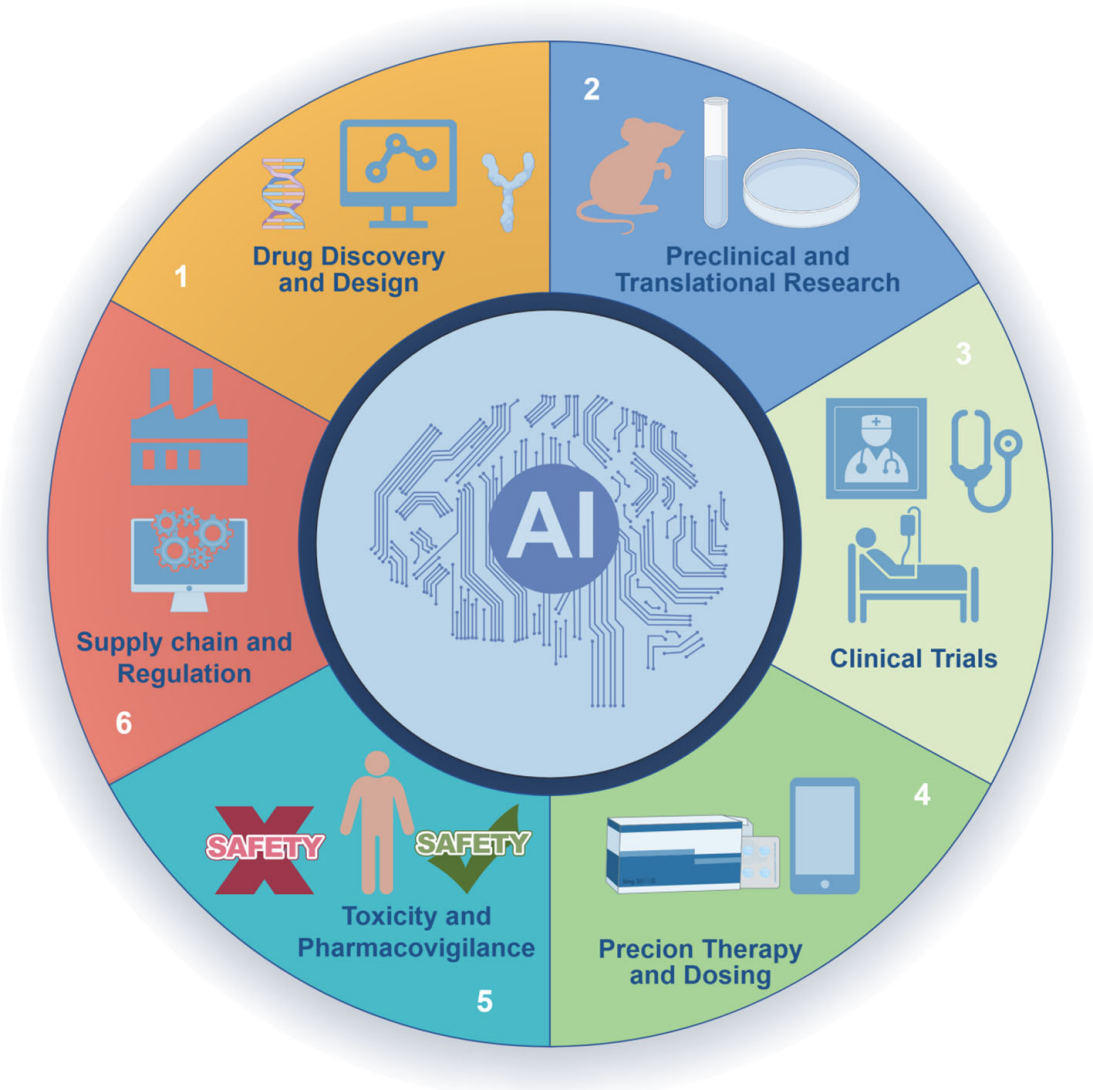
AI across the oncology drug lifecycle. A circular schematic depicts six stages: **(1)** Drug discovery and design; **(2)** Preclinical and translational research; **(3)** Clinical trials; **(4)** Precision therapy and dosing; **(5)** Toxicity and pharmacovigilance; **(6)** Supply chain and regulation. The central “AI” hub emphasizes cross-cutting methodsthat integrate evidence across stages.

Oncology drug development encompasses a comprehensive process, ranging from target discovery and compound screening to clinical trial design. In recent years, AI technologies have been progressively integrated into every stage of this pipeline, catalyzing a paradigm shift in drug discovery and development. Consistent with **Figure 1**, we begin with discovery and design (Sections 3.1–3.2) and highlight preclinical/translation utilities (knowledge-graph mechanism hypotheses, multi-omics triangulation) that connect discovery to clinical trials (Section 3.3).

### 3.1 Target discovery and validation

Drug target identification represents the initial and critical step of new drug development. Given the intricate pathogenic mechanisms of cancer, researchers are increasingly employing AI—particularly deep learning techniques—to mine vast biological datasets for novel therapeutic targets. By integrating multi-omics data, including genomics, transcriptomics, and proteomics, AI can uncover key molecular networks and vulnerable nodes driving tumor progression (12, 23). Machine learning models can correlate genomic mutations, expression profiles, and clinical outcomes to identify subtype-specific oncogenic drivers or signaling pathways. Compared to manual analysis, AI excels at handling the immense scale and complexity of biological networks, revealing patterns and associations that may elude human experts (24, 25). Advanced AI models such as AlphaFold have further revolutionized target validation (26, 27). AlphaFold predicts the three-dimensional structures of proteins with remarkable accuracy, providing crucial insights into protein conformations and druggability (28, 29). Researchers can now assess whether a protein contains ligand-binding pockets suitable for small-molecule intervention, thus determining its potential as a viable drug target (29, 30). In essence, AI has enabled transformative progress from the macroscopic level of omics networks to the microscopic resolution of molecular structure, thereby accelerating the early phases of oncology drug development.

Beyond target discovery, AI also contributes to target validation by assessing the causal relationship between targets and disease, as well as their druggability. Traditional validation relies heavily on *in vitro* and animal models, which are time-consuming and resource-intensive. Today, researchers are leveraging AI to construct disease knowledge graphs and causal inference models for target validation. By embedding molecular interaction data from literature and databases into graph-based frameworks, AI can simulate the therapeutic effect of inhibiting a specific target and infer possible mechanisms of action (1, 31, 32). For instance, studies have integrated drug–disease and drug–target networks and employed deep learning models (e.g., DeepDR) to extract high-level features through random walk strategies, predicting drug efficacy against particular targets and cancers (1) These network-based AI approaches enable system-level validation of target perturbation effects, thereby enhancing the reliability of target selection. Overall, AI facilitates more efficient (faster identification of critical targets) and more precise (elimination of false positives) discovery and validation processes, thereby increasing the success rate of oncology drug development from the outset. Accurate protein structures (e.g., AlphaFold) inform pocket assessment and hypothesis-driven design for oncology targets; however, prospective studies directly linking structure prediction to improved oncology clinical outcomes remain limited.

### 3.2 Virtual drug screening and design

Once a target is identified, the next step involves discovering compounds capable of modulating the target. Traditional high-throughput screening (HTS) requires the synthesis and testing of thousands of molecules, which is both time-intensive and costly. The advent of AI has brought transformative improvements to *in silico* drug screening. Deep-learning pipelines enable in-silico screening, design, and optimization of large libraries, expediting early discovery.

One prominent strategy is the use of generative models such as generative adversarial networks (GANs) and reinforcement learning-based molecule generators (33). These models encode molecules as SMILES strings or molecular graphs, and after neural network training, they can generate entirely new chemical structures (31, 34). Generative models can be directed to create compounds with desired properties—for example, molecules with high affinity for a specific target and favorable pharmacokinetic profiles. Compared to random screening, AI-generated compounds are more targeted, significantly improving hit rates. Studies have shown that reinforcement learning can continuously optimize generation strategies to produce compounds with enhanced activity and drug-likeness, reducing early-stage design timelines from months to weeks (31, 35). For instance, algorithms like ReLEASE use reinforcement learning to iteratively evolve molecular structures, generating novel compounds with activity against predefined proteins (36). These AI-generated libraries have opened expansive chemical spaces for cancer drug discovery.

In addition to *de novo* design, AI is also used to virtually screen existing large-scale compound libraries for potential drug candidates. Deep learning models can predict binding affinity and bioactivity between compounds and biological targets by learning from molecular structures and target features (37). Graph neural networks (GNNs), for example, treat molecules as graph-based structures and efficiently model the relationship between structure and activity, enabling rapid screening of millions of compounds (38–40). Compared to traditional docking methods, AI models offer superior speed and accuracy, rapidly narrowing down promising leads. Reports suggest that AI-driven virtual screening can shorten early discovery cycles from months to weeks, particularly impactful in oncology where rapid options are needed. Furthermore, researchers have combined molecular dynamics simulations with machine learning to refine predictions of ligand–target interactions. AI can analyze trajectory data to evaluate the stability of ligand–protein complexes, filtering out initially promising but potentially unstable compounds (40, 41). This hybrid “simulation-plus-learning” approach further enhances screening precision.

In summary, AI has greatly improved the efficiency of lead compound discovery in oncology. It enables the innovative design of novel molecules, high-throughput screening of existing libraries, and optimization of drug-like properties. These advances reduce experimental workload and cost, accelerating the progression of candidates into preclinical development. Studies have shown that AI-based molecular screening and design significantly shorten development timelines and reduce expenses (42), offering faster and more diverse therapeutic options for cancer patients.

### 3.3 Clinical trial optimization

Following the preclinical stage, the design and execution of clinical trials are pivotal for the successful translation of oncology drugs into clinical use. Traditional clinical trials often suffer from challenges such as slow patient recruitment, prolonged timelines, and low success rates. AI technologies are now offering innovative solutions to optimize various aspects of clinical trial operations.

First, AI enhances patient stratification and recruitment—crucial steps in trial initiation. Natural language processing (NLP), a subfield of AI, can be used to automatically extract relevant patient characteristics from electronic health records (EHRs) and clinical narratives to identify individuals eligible for trial enrollment (43, 44). Previously, this process relied heavily on manual chart review, which is time-consuming and prone to missing eligible participants. Modern NLP algorithms are capable of parsing large volumes of unstructured clinical texts—such as physician notes, pathology reports, and diagnostic summaries—to extract key fields like diagnoses, biomarker status, and treatment history (45). For example, in lung cancer clinical trials, NLP tools can screen hospital databases to identify patients harboring specific gene mutations, thereby accelerating recruitment. Beyond eligibility screening, machine learning models can stratify patients into subgroups based on multi-dimensional data and predict which subsets are more likely to benefit from investigational therapies. This stratified approach allows for biomarker-driven trial design, improving signal detection and statistical power (46). Such “AI-enabled screening and enrichment” enhances both the efficiency and success probability of clinical trials. Compliance note. In all examples, EHR-based NLP screening occurs within covered entities under IRB approval and data-use agreements; no external re-identification of individuals is performed.

Second, AI can assist in adaptive trial design and real-time monitoring. Traditional randomized controlled trials (RCTs) with fixed protocols may be inefficient under certain conditions. In contrast, adaptive clinical trials, which adjust trial parameters based on interim results, offer a more flexible and efficient approach. AI models are well-suited for real-time analysis of accumulating trial data, providing support for dynamic decision-making (46, 47). For instance, predictive models can leverage historical and preliminary data to adjust dosing regimens or sample sizes mid-trial, ensuring patients receive more effective dosages or shortening trial duration (47). Additionally, AI enables the development of novel concepts such as synthetic control arms and digital twins (48). A synthetic control arm uses historical real-world data and AI modeling to simulate outcomes for control patients, thus reducing the need for placebo or standard-of-care enrollment in interventional trials (49). This approach is particularly valuable in oncology, where ethical and practical concerns favor maximizing access to new therapies. Digital twins—virtual representations of individual patients constructed using clinical and biological data—allow for simulated comparisons of different treatment outcomes and provide in-trial decision support (50). These techniques have the potential to not only enhance trial efficiency but also extend evidence generation beyond conventional settings.

Finally, AI contributes to safety monitoring and adherence management during clinical trials. Risks include selection bias or manipulation in algorithmic enrollment and privacy concerns when handling sensitive records. Pragmatic safeguards include pre-specified enrollment algorithms with version control, independent DSMB oversight, and auditable logs of model versions and inclusion/exclusion decisions; data handling should be IRB-approved and HIPAA-compliant with de-identification and role-based access governed by data-use agreements.

Timely identification of adverse events (AEs) and data anomalies is critical for ensuring patient safety. Machine learning models can continuously analyze incoming trial data to detect abnormal physiological patterns or early signs of AEs and trigger alerts for clinical intervention. For instance, AI-based systems have been used to monitor patient-reported outcomes and wearable device data, enabling early detection of serious side effects and mitigating risks through timely action.

In summary, AI is increasingly empowering all facets of clinical trial optimization (51). From patient identification and stratification to adaptive protocol design and continuous monitoring, AI tools are reshaping how trials are conducted. Literature reports indicate that AI can substantially reduce the cost and duration of trials while improving the likelihood of regulatory approval (37). In the context of oncology—where innovation is both urgent and competitive—AI-driven clinical trial optimization offers a pathway to bring effective therapies to patients more rapidly and efficiently. Representative AI applications, data sources, and evidence types are summarized in **Table 1**.

**Table 1:** Representative AI applications in oncology drug development and management: tasks, exemplar methods, data sources, and evidence types.

Lifecycle stage	Task	Exemplar AI method/tool	Oncology use case (illustrative)	Primary data source(s)	Evidence type in cited studies	Representative refs
Discovery & design	Structure/target assessment	AlphaFold 2/3 structure prediction	Pocket/complex modeling informs target druggability	Protein structures (PDB), sequences	Structural confidence/coverage (pLDDT/lDDT); qualitative enablement	(26, 30)
Discovery & design	*De novo* molecular design	Graph-based generative models, transformers	Novel chemotypes for oncology targets	Public/enterprise chemical corpora	Retrospective hit enrichment; faster ideation cycles	(33–38)
Discovery & design	Virtual screening/prioritization	GNN/DL rescoring; multi-task QSAR	Prioritize candidates for wet-lab validation	Assay libraries, docking outputs	Improved enrichment vs. baseline docking (context-dependent)	(37–40)
Preclinical/translation	Mechanism-informed hypothesis generation	Knowledge graphs + link prediction	Target–compound–disease triangulation	Literature/omics/chemical DBs	Qualitative prioritization vs. manual curation	(40)
Clinical trials	Eligibility screening/recruitment	EHR NLP + rules/phenomapping	Faster identification of eligible patients	EHR notes + structured fields	↑ screening efficiency; potential ↑ power	(43–46)
Clinical trials	Control arm strategies	Synthetic controls/digital twins	Augment/replace control cohorts	Historical trials, registries, EHR	Potential sample-size reduction; faster decisions	(49, 50)
Precision therapy & dosing	Dose personalization	CURATE.AI platform	Individualized dosing in solid tumors (feasibility)	Longitudinal labs/response	Feasibility; individualized dose trajectories	(56)
Resistance & combinations	Synergy prediction	Knowledge-graph embeddings; DL/GNN synergy models	Rational combination design to delay resistance	Pharmacogenomic & assay datasets	Retrospective synergy ranking; preclinical validation	(57–60)
Toxicity & pharmacovigilance	AE extraction (EHR)	EHR-NLP pipelines	irAE monitoring in oncology	EHR clinical notes	AE frequencies concordant with trial reports	(73)
Toxicity & pharmacovigilance	Signal fusion	FAERS/VigiBase + EHR-NLP	Earlier/more sensitive signal detection	Spontaneous reports + EHR	Earlier detection in fusion settings (qualitative)	(73)
Supply chain & regulation	Anti-counterfeiting/traceability	Blockchain + anomaly detection	Serialization and track-and-trace	Logistics/serialization streams	Qualitative improvements in traceability	(70–72)

## 4 AI-driven precision medication management in oncology

With the advent of increasingly diverse anticancer therapeutics, the challenge of tailoring treatment strategies to individual patients has become a central focus in clinical oncology. AI plays a pivotal role in enabling precision medication, including optimizing personalized treatment plans, predicting drug resistance, and managing toxicity.

### 4.1 Personalized treatment decision-making

Each cancer patient exhibits a unique combination of genetic background, tumor molecular profile, and overall health status, resulting in highly variable responses to therapy. AI provides powerful tools to facilitate individualized medication strategies. By integrating patient-level data such as genomic mutations, gene expression profiles, pathophysiological parameters, and clinical history, machine learning models can predict a patient’s likelihood of responding to specific drugs, thereby supporting clinicians in selecting the most suitable regime**n** (52). For example, in advanced cancer cases where multiple treatment options are available, AI models can calculate the expected efficacy and risk for each therapy based on outcomes from similar patient cohorts, offering quantitative insights for decision-making.

Studies have demonstrated that multi-omics data, when input into AI models, can reveal biomarkers associated with drug response or tolerability (53). These biomarkers enable models to predict whether a patient is likely to benefit from a given treatment (54). In one multicenter study, an AI model incorporating tumor mutational profiles, transcriptomics, and clinical features outperformed traditional biomarkers such as PD-L1 expression in predicting response to immune checkpoint inhibitors. Such tools facilitate multidisciplinary team (MDT) discussions by providing data-driven guidance to design tailored treatment regimens, thereby realizing the promise of precision oncology.

Beyond treatment selection, AI also contributes to dose optimization. Traditional dosing schemes often follow fixed schedules or body surface area-based formulas, which inadequately account for individual variability. AI techniques—particularly reinforcement learning—can dynamically adjust drug dosage or scheduling based on real-time patient response, maximizing efficacy while minimizing toxicity (55). For instance, researchers have trained deep reinforcement learning models using virtual patient simulations to learn adaptive dosing strategies that prolong tumor control without exacerbating side effects. In real-world scenarios, AI-assisted dose personalization platforms such as CURATE.AI exemplify this approach: by analyzing a patient’s own longitudinal data, the platform continuously recommends individualized dosage adjustments (56). In a pancreatic cancer study, CURATE.AI successfully optimized dosing to reduce tumor markers while maintaining tolerable toxicity levels. These applications demonstrate that AI can break away from the “one-size-fits-all” paradigm of maximum tolerated dose (MTD), identifying optimal therapeutic windows tailored to each patient. As intelligent dosing systems become more widely adopted, personalized treatment is expected to achieve both improved outcomes and enhanced safety profiles.

### 4.2 Resistance prediction and combination therapy design

Modern pharmacovigilance increasingly adopts a data-fusion paradigm in oncology, combining FAERS/VigiBase disproportionality with EHR-NLP extraction of adverse events to enable earlier, more sensitive signal detection. The adaptive evolution of cancer cells frequently leads to therapeutic resistance—one of the major causes of treatment failure in oncology (17). Studies show EHR-NLP can recover immune-related AE frequencies concordant with trial reports, supporting its use to supplement spontaneous reporting systems (57). To enable early intervention, researchers have begun applying AI models to predict the onset of resistance and propose counterstrategies. One approach involves training time-series models (e.g., recurrent neural networks or Bayesian networks) on longitudinal data such as tumor burden changes and imaging features to anticipate progression. Other studies utilize sequential genomic data from repeated biopsies to reconstruct evolutionary trajectories and detect emerging resistant clones. AI can identify mutation patterns or expression signatures associated with resistance and issue alerts when similar trends are detected, allowing clinicians to consider preemptive treatment adjustments or initiate combination therapy.

Combination therapy is a key strategy to overcome resistance by simultaneously targeting multiple oncogenic pathways and reducing the likelihood of tumor escape. Traditionally, identifying effective drug combinations was a laborious and largely empirical process. AI has introduced a data-driven framework for discovering synergistic drug pairs by integrating knowledge graphs and deep learning. By constructing multi-relational graphs encompassing drugs, genes, and diseases, AI models can mine for combinations with predicted synergistic potential. For instance, the knowledge graph embedding model KGE-DC has been used to predict drug pairs with enhanced efficacy against specific cancers, several of which have been validated in preclinical models (58). Deep learning models can also predict drug synergy by integrating compound structures, target information, and cellular assay data (59). Graph neural networks trained on large pharmacogenomic datasets have successfully ranked drug pairs for their synergy potential in specific tumor types and have retrospectively identified many clinically validated combinations (60, 61). Such AI-enabled screening transforms combination therapy development from trial-and-error to evidence-based selection.

Importantly, these models can also incorporate known resistance mechanisms. When a resistance pathway is activated, AI can recommend co-administration of agents that inhibit compensatory signaling nodes. For instance, in non-small cell lung cancer with resistance to EGFR inhibitors, AI-guided knowledge graphs may suggest addition of a bypass pathway inhibitor to restore tumor control (62). As AI continues to evolve in this domain, it is expected to drive the development of rational combination regimens that effectively delay or overcome resistance, thereby improving long-term cancer control.

### 4.3 Toxicity prediction and management

While anticancer agents offer therapeutic benefits, they are frequently accompanied by considerable toxicity. Predicting and managing these adverse effects is a vital component of precision oncology. AI offers novel solutions in this domain.

On one hand, AI can be used to forecast adverse drug reactions (ADRs). Machine learning models trained on historical clinical trial data and real-world pharmacovigilance databases can learn associations between drug regimens, molecular properties, and specific toxicities (22). For example, the CT-ADE database, which includes over 2,500 drugs and more than 160,000 drug–adverse event pairs, has been used to train large language models that predict ADR risk across various doses and patient profiles (22). Incorporating individual characteristics—such as age, comorbidities, and genetic polymorphisms—enhances predictive accuracy. In oncology, these tools enable clinicians to identify high-risk patients prior to treatment initiation, allowing for proactive mitigation strategies such as dose modification or preventive monitoring. For instance, if an AI model predicts a high likelihood of severe myelosuppression from a chemotherapy regimen in a specific patient, the clinician might consider alternative treatments or supportive interventions.

On the other hand, NLP techniques empower real-time monitoring of toxicity from unstructured clinical data. Many ADRs are documented in free-text formats such as physician notes or discharge summaries. NLP algorithms can extract these data to facilitate near real-time pharmacovigilance. For example, studies have demonstrated that NLP can accurately capture immune-related adverse events from EHRs of hospitalized oncology patients, with extracted frequencies closely aligning with those reported in trials (63). When datasets are limited, transfer learning from related oncology cohorts, temporally held-out (“future”) validation, and external replication across sites should be used to mitigate overfitting and quantify generalizability. Such systems can be embedded in hospital safety monitoring frameworks to trigger alerts for severe events, enabling timely clinical response. Moreover, regulatory agencies have begun utilizing NLP to analyze patient-reported symptoms on social media and online forums, potentially detecting rare or overlooked side effects. This AI-driven approach to pharmacovigilance ensures a more comprehensive and proactive safety net for cancer therapies (64).

Finally, AI supports dose adjustment and supportive care in response to emerging toxicities. Traditionally, toxicity management relies on clinical experience, which may vary in consistency and timeliness. AI-powered systems can analyze the progression of adverse reactions and suggest evidence-based interventions. For example, in cases of Grade 3 neutropenia, AI models may recommend delaying the next treatment cycle or initiating granulocyte-colony stimulating factor (G-CSF) prophylaxis based on projected recovery and infection risk (65, 66). These decision support tools enhance the standardization and responsiveness of toxicity management, potentially preventing escalation to life-threatening complications.

In summary, AI is enabling a shift toward proactive and individualized toxicity management in oncology. By predicting high-risk scenarios, mining toxicity signals, and guiding patient-specific interventions, AI promises to minimize treatment-related harm and ensure safer therapeutic journeys for patients.

## 5 The role of AI in oncology drug supply chain and regulatory oversight

The management of oncology drugs extends beyond research and clinical application to include the production, distribution, and post-marketing regulatory monitoring. AI has begun to play an increasingly important role in optimizing pharmaceutical supply chains and enhancing pharmacovigilance and drug reevaluation. A comparative view of conventional versus AI-enhanced workflows across the lifecycle is provided in **Table 2**.

**Table 2:** Conventional versus AI-enhanced workflows across the oncology drug lifecycle.

Pipeline step	Traditional workflow	AI-enhanced workflow	Typical AI inputs	Indicative benefit (from cited studies)	Risks/considerations	Mitigations/good practice	Representative refs
Structure/target triage	Manual homology modeling; slow validation	AlphaFold-enabled triage + knowledge-graph context	Sequences, structures, KG edges	↑ structure coverage; faster triage/hypothesis generation	Over-interpreting confidence	Combine confidence with orthogonal evidence	(26, 30)
Library design & screening	Rule-based QSAR/docking	Generative design + GNN screening + active learning	Chem corpora; assay labels	Fewer design cycles; months→weeks (tempered)	Dataset bias; implausible chemotypes	Domain constraints; external test sets	(33–38)
Trial recruitment	Manual chart review	EHR NLP eligibility + phenomapping	Notes + structured EHR	↑ screening efficiency; potential ↑ power	Privacy; portability across sites	IRB/DUA; site calibration	(43–46)
Control arm strategy	Conventional RCT control only	Synthetic controls/digital twins	Registries; historical trials; EHR	Potential ↓ control-arm size; quicker decisions	Exchangeability assumptions	Pre-specification; sensitivity analyses	(49, 50)
Dose selection	Fixed/weight-based dosing	CURATE.AI individualized dosing	Longitudinal response series	Personalized dose trajectories; feasibility	Overfitting to noise	Temporal validation; guardrails	(56)
Post-market safety	SRS disproportionality alone	FAERS/VigiBase + EHR-NLP fusion	Spontaneous reports + EHR notes	Earlier/rarer signal detection; complements under-reporting	Confounding; duplicates	De-duplication; clinician adjudication	(73)
Supply chain integrity	Paper/legacy serialization	Blockchain + anomaly detection	Serialization and scan logs	Improved traceability; counterfeit deterrence	Interoperability, cost	Standards; pilot roll-out	(70–72)

### 5.1 Optimization of the oncology drug supply chain

Anticancer drugs are typically expensive and involve complex supply chains encompassing raw material procurement, manufacturing, warehousing, and delivery to hospitals and pharmacies. AI, through predictive analytics and intelligent scheduling, can significantly improve the efficiency, transparency, and reliability of the supply chain to ensure timely and sufficient drug availability.

In terms of demand forecasting, AI algorithms can integrate various determinants—historical consumption, seasonal patterns, epidemiological trends, and healthcare policy shifts—to improve the accuracy of drug usage predictions (67). Traditional methods often rely solely on linear projections of historical sales data, whereas AI models can analyze hundreds or thousands of variables simultaneously, capturing latent correlations. For instance, a major pharmacy chain employing AI-based forecasting reduced prediction errors by 20–30% and decreased stock-out incidents by 15%. Similarly, a hospital inventory management system using AI to factor in seasonal disease trends, regional demographics, and prescribing habits achieved a forecasting accuracy of 94%, outperforming traditional models (78%) (67). Accurate demand forecasting enables manufacturers and providers to optimize production schedules and inventory levels—preventing shortages that could endanger patients and avoiding waste due to overstock or expiry. For oncology drugs, such precision is particularly critical, as treatment delays due to drug shortages can have serious consequences, while expired drugs represent substantial economic losses. AI helps match supply to demand with high fidelity, ensuring uninterrupted access to critical medications.

Furthermore, AI in combination with Internet of Things (IoT) technologies enhances logistics and warehouse management. AI-powered dispatch systems can optimize delivery routes in real time based on traffic, weather, and urgency of demand at different locations, thereby ensuring efficient transportation and cold chain integrity (68) During public health emergencies or natural disasters, AI systems can rapidly replan routes to prioritize delivery of life-saving oncology drugs. In warehousing, AI algorithms optimize stock rotation based on expiry dates and consumption rates, enabling timely redistribution of surplus inventory to high-demand areas and reducing wastage. One report showed that a hospital network reduced inventory holding costs by 23% and emergency restocking events by 35% after implementing AI-based inventory strategies (69, 70) These improvements are particularly impactful for high-cost oncology drugs, contributing to both cost-effectiveness and timely access for patients.

A particularly notable advancement is the integration of AI with blockchain technology to enhance drug authenticity and traceability. Counterfeit and illegally circulated medications pose serious risks to patient safety globally. Blockchain provides an immutable distributed ledger for tracking every stage of a drug’s journey—from manufacturing to dispensing. When combined with AI for real-time data analysis, these systems can effectively detect anomalies and combat counterfeit products. Pilot programs in some countries have leveraged blockchain to log logistics data of anticancer drugs, while AI-based image recognition verifies packaging and serial codes (71, 72). Any discrepancies in shipping records or packaging features trigger AI-generated alerts. One such system helped authorities intercept counterfeit oncology drugs worth millions within six months (73). By identifying subtle differences in barcode patterns or packaging design and cross-verifying with blockchain records, the system significantly improved inspection accuracy. Altogether, the synergy of AI and blockchain enables intelligent supply chain management—from predictive distribution to secure authentication—ultimately safeguarding access and safety for cancer patients.

### 5.2 Pharmacovigilance and drug re-evaluation

Post-marketing surveillance and real-world evidence (RWE) evaluation in oncology primarily addresses clinical safety and effectiveness in routine care, leveraging spontaneous reporting systems (e.g., FAERS/VigiBase) and EHR/claims rather than economic outcomes. Studies are conducted by public agencies and industry under regulatory oversight, using methods aligned with pharmacovigilance and RWE standards (57). AI supports this process by enhancing case ascertainment (e.g., EHR-NLP adverse event extraction) and by fusing SRS with EHR/claims to improve sensitivity for earlier signal detection. Insights from post-market signals and RWE can inform indication refinement and drug repurposing, closing the loop with upstream discovery and trial design.

AI significantly enhances the sensitivity and efficiency of AE signal detection. Large spontaneous reporting systems, such as the FDA Adverse Event Reporting System (FAERS) and WHO’s VigiBase, receive enormous volumes of suspected ADR reports annually. Here, “high-risk signal” denotes a serious or unexpected adverse-event association that crosses predefined thresholds (e.g., disproportionality metrics) in spontaneous-reporting or EHR-derived analyses and warrants regulatory or clinical action.Traditional workflows—manual case review and single-metric disproportionality (e.g., PRR/ROR) applied to sparse, heterogeneous reports—may miss early or low-frequency patterns; multi-source fusion of FAERS/VigiBase with EHR-based NLP helps address under-reporting and improves sensitivity (see existing citations in this paragraph).

AI algorithms are adept at mining such large datasets to identify statistically significant associations—potential safety signals. Machine learning models can estimate background incidence rates for specific AEs in the general population and compare them with observed reporting rates associated with particular drugs, highlighting unusual patterns (73). Some AI-based systems have demonstrated the ability to detect rare but serious ADRs earlier than traditional methods. For example, while post-market reports of cardiac dysfunction associated with a newly approved cancer drug were initially scattered across different countries and went unnoticed by manual review, an AI model aggregated and flagged these cases promptly, prompting regulatory warnings. Moreover, as discussed previously, NLP techniques can extract AE data from non-traditional sources such as clinical notes and social media. Studies have shown that frequencies of chemotherapy-related AEs extracted via NLP from electronic health records closely match clinical trial reports, validating their use in supplementing official systems Hence, modern pharmacovigilance is evolving toward a “data fusion” model—AI integrates spontaneous reports, literature, EHRs, and patient-generated data to provide multidimensional safety monitoring. This comprehensive approach enables earlier detection and more effective risk mitigation.

In the case of high-risk, high-cost oncology drugs, regulatory agencies are increasingly emphasizing the importance of RWE for long-term effectiveness and safety evaluation. AI plays a crucial role in RWE analysis, which often involves large, heterogeneous datasets from EHRs, insurance claims, biobanks, and patient registries. AI models can synthesize fragmented data sources to assess drug performance across broader populations and extended timeframes. For example, national cancer registry and insurance claims data analyzed by AI can reveal whether a new targeted therapy confers survival benefits in elderly patients or those with multiple comorbidities—groups underrepresented in clinical trials. Moreover, AI can detect late-onset toxicities, such as secondary malignancies years after treatment, which are typically missed in trial settings [. Causal inference models powered by AI can approximate randomized controlled trial conditions in observational data. For instance, AI-based matching techniques can construct control cohorts similar to treatment arms to estimate the causal effect of immunotherapy on long-term survival. While observational studies have inherent limitations, AI improves confounding control and result robustness, supporting regulatory decisions regarding label expansion or indication refinement.

Additionally, AI is transforming drug repurposing and post-market efficacy reevaluation. Many approved drugs for non-oncological indications may have unrecognized anticancer potential. AI can correlate drug-induced multi-omics signatures with tumor molecular characteristics to identify new therapeutic applications. One such model suggested that a legacy drug could suppress oncogenic pathways in a specific tumor subtype—a prediction later validated in clinical studies, illustrating AI’s value in uncovering hidden efficacy (74). For already approved oncology drugs, AI can continuously monitor real-world outcomes and identify subgroups with differential efficacy. If real-world data indicate suboptimal outcomes in certain genotypes, regulators may revise usage guidelines accordingly. In the future, drug approval may transition from a “one-time decision” model to a dynamic, AI-informed regulatory approach, with indications and usage evolving based on real-time evidence.

In conclusion, AI is facilitating a shift in drug regulation from reactive to proactive. From real-time AE surveillance to dynamic benefit-risk assessment, AI empowers regulators to detect issues earlier and base decisions on more comprehensive evidence. In the high-stakes context of oncology therapeutics, AI-driven regulatory oversight ensures that innovative treatments deliver maximum benefit with minimal harm.

## 6 Challenges and future directions

Despite remarkable progress in the application of AI to oncology drug management, fully realizing its transformative potential requires addressing a number of critical challenges. These span technical limitations, clinical translation barriers, as well as ethical and regulatory concerns.

### 6.1 Technical challenges

Cancer-related data are inherently heterogeneous, encompassing genomic sequences, medical imaging, pathology slides, electronic health record (EHR) text, and data from wearable devices. Effectively integrating such multimodal data remains one of the core technical challenges in AI development. Single-modality data often fail to comprehensively characterize the disease state, whereas multimodal fusion promises a more holistic understanding. However, disparities in scale, format, and quality between modalities can lead to a “data gap” problem. Current approaches often employ deep learning models to extract features from each modality separately, which are then integrated at a higher level—for instance, combining convolutional neural network (CNN) features from imaging with fully connected representations from genomics to predict treatment response or prognosis (75). Although early results are promising, training robust multimodal models requires large paired datasets and careful architectural design to avoid modality dominance and information imbalance.

Advances in technologies such as federated learning offer potential solutions by enabling multi-institutional collaboration without compromising data privacy (76). This allows for distributed training of AI models across centers, pooling insights from rare cases or underrepresented populations without centralizing data. Success in multimodal fusion would empower AI systems to consider cancer from multiple biological and clinical dimensions, generating more accurate and context-aware recommendations.

Although data-rich, the biomedical domain often suffers from limited high-quality labeled data. Clinical trial datasets are typically small and expensive to acquire, and data on rare cancers or adverse events are especially scarce. This creates challenges for traditional deep learning, which relies on large training sets for robust performance. In response, researchers are exploring techniques such as transfer learning and few-shot learning. In transfer learning, models are pre-trained on large datasets (e.g., from related cancer types or synthetic data) and fine-tuned on smaller target datasets, enhancing generalizability. Data augmentation techniques, such as generative adversarial networks (GANs) that synthesize pathology images, are also being adopted (77, 78). Recent studies have demonstrated that pre-trained models can perform reliably even with as few as dozens of patients in the fine-tuning phase (79). Nevertheless, risks remain: overfitting, data drift, and learning from noise rather than signal can impair model reliability. Addressing the small-sample challenge is essential for expanding AI into understudied cancers and rare toxicities.

A further concern is the “black box” nature of many high-performance AI models, particularly deep learning systems, which lack interpretability (80).To move from promise to practice, we outline pragmatic mitigations mapped to common failure points in translational deployment. For external validity and model drift, teams should pre-register model-update rules, evaluate with temporal hold-out or rolling-origin splits, and monitor data and performance drift with predefined review triggers. For fairness, subgroup performance audits across clinically relevant and protected attributes should be routine, with corrective re-weighting, threshold adjustment, or group-wise calibration and explicit reporting of disparity metrics. For prospective evidence, embedding models in randomized or stepped-wedge/pragmatic trials with patient-important outcomes and decision-impact endpoints is essential. For explainability, case-level explanations (e.g., SHAP) and knowledge-anchored rationales, coupled with clinically sensible feature sets and clinician-centered UI pilots, can improve trust. Finally, for regulatory alignment, SaMD-style change-control plans, versioning and auditable logs, and post-market algorithmovigilance using real-world data with performance floors and remediation pathways should be adopted.

In clinical contexts, both physicians and patients often seek to understand the rationale behind AI-generated recommendations. Lack of transparency may undermine trust and hinder adoption. Enhancing explainability has thus become a central goal in medical AI. Several strategies have emerged: (1) developing inherently interpretable models (e.g., decision trees or knowledge graph-based reasoning), which may sacrifice some accuracy for greater transparency (81, 82),(2) applying *post hoc* explanation methods, such as attention mechanisms, SHAP values, or counterfactual reasoning, to identify key features driving prediction (83), (3) integrating domain knowledge into model architecture or outputs, anchoring AI reasoning to established medical principles and improving credibility (84). Explainability not only enhances clinical trust but also facilitates new scientific discovery by highlighting previously unrecognized patterns. As regulatory frameworks increasingly require some level of interpretability, explainable AI will be pivotal for integrating AI into routine medical practice.

### 6.2 Challenges in clinical translation

Many AI models demonstrate high performance in retrospective datasets, yet lack prospective validation through clinical trials—a significant barrier to clinical adoption. Most current evidence for AI’s effectiveness in medicine derives from retrospective analyses rather than randomized controlled trials (RCTs), the gold standard for assessing intervention outcomes (85). Without prospective validation, clinicians may remain skeptical of AI-driven recommendations. It is therefore imperative to design trials that evaluate whether AI tools improve clinical decision-making, treatment outcomes, or cost-effectiveness. In some domains, such as radiology, prospective studies have already shown that AI can enhance diagnostic accuracy. Similarly, oncology-focused trials are needed to assess whether AI-guided treatment recommendations translate into better survival, reduced toxicity, or improved quality of life.

The successful integration of AI into clinical workflows also hinges on physician acceptance and usability. If medical professionals are unfamiliar with or distrustful of AI tools, their potential will remain untapped. Surveys indicate that many clinicians are hesitant due to limited understanding of AI, concerns over reduced autonomy, or fear of misinterpretation. Therefore, it is critical to provide AI education and training in medical curricula and continuous professional development program (43). Equally important is designing AI systems that align with existing clinical pathways, defining clear roles for AI (e.g., pre-screening, suggesting alternative treatments) while preserving clinician authority in final decision-making. Early adopter experiences suggest that AI-augmented multidisciplinary team (MDT) discussions can improve the speed and comprehensiveness of treatment planning—provided that communication and expectations are well-managed. Over time, as younger clinicians grow up with AI as a standard tool, we may see the emergence of a new norm: the AI-empowered physician.

### 6.3 Ethical and regulatory considerations

AI systems in healthcare depend heavily on sensitive patient data, raising significant concerns regarding privacy and data security. Genomic information, clinical histories, and EHRs contain personal health identifiers, and improper use or breaches could have serious consequences. Regulations such as the General Data Protection Regulation (GDPR) impose strict requirements on the collection, processing, and storage of such data. AI developers must implement safeguards, including de-identification, encryption, and transparent consent protocols. Federated learning offers a privacy-preserving solution by training models locally at each institution and aggregating only model parameters—ensuring that data remain decentralized and secure (86). This approach has already been piloted in multi-center cancer prognostic modeling. In the future, privacy-preserving technologies such as differential privacy and blockchain-based audit trails may further enhance AI system transparency and accountability.

Bias and fairness are equally critical concerns. AI models trained on unbalanced or non-representative datasets risk perpetuating or amplifying existing health disparities. For instance, an oncology model developed primarily using data from Western populations may perform poorly in other ethnic groups, potentially leading to misdiagnoses or inappropriate recommendations. Several studies have highlighted biases in medical AI due to sample selection and measurement errors (87, 88). Developers must strive for inclusive training datasets that reflect population diversity, and where data are sparse, models should incorporate uncertainty estimates or reject predictions. Fairness metrics—such as subgroup performance comparison—should be routinely applied to detect and mitigate biases. AI in medicine must uphold the principles of equity and justice: it should benefit all patients, not just privileged groups (89). Organizations such as ASCO have proposed ethical guidelines for responsible AI development, emphasizing transparency, fairness, and patient-centeredness (90). Regulatory agencies must also screen AI systems for potential bias before approval, ensuring that no vulnerable populations are adversely affected.

Finally, the regulatory frameworks for AI in healthcare remain nascent. Most jurisdictions are still evolving their approaches to software as a medical device (SaMD), especially for adaptive AI systems. Key questions include: How is safety and efficacy demonstrated? How are updates and version changes monitored? Who is liable in the event of errors? Agencies such as the FDA have proposed frameworks requiring algorithm change protocols and ongoing performance monitoring. Future policies may mandate transparency reports, explanation of model logic, and continuous post-market surveillance—a concept termed “algorithmovigilance” (91, 92). AI systems may eventually be held to standards akin to pharmacovigilance, with systematic monitoring and recall mechanisms. As AI gains autonomy, the question of responsibility will become more complex. While current regulations place ultimate responsibility on licensed clinicians, new frameworks may need to clarify shared accountability between AI developers, providers, and users. The ideal regulatory approach must strike a balance: ensuring safety and accountability without stifling innovation. Such a balanced framework will require close collaboration among regulators, clinicians, researchers, and industry stakeholders.

## 7 Conclusion

AI is driving a profound paradigm shift in oncology drug management. From drug discovery and development to clinical application and post-marketing surveillance, AI offers unprecedented gains in efficiency and decision-making support. As discussed above, AI facilitates more precise identification of drug targets and reduces development costs; it transforms empirical drug discovery into a data-driven process through virtual screening and molecular design; in clinical trials, AI enhances patient selection and trial design, improving trial success rates; during clinical application, AI enables personalized treatment plans and dynamic dosing strategies tailored to each patient’s unique profile; it addresses key challenges such as drug resistance and toxicity through early prediction, combination therapy design, and proactive management of adverse effects; within pharmaceutical supply chains, AI ensures efficient, secure drug distribution through demand forecasting, anti-counterfeiting, and logistics optimization; in regulatory contexts, AI supports real-time pharmacovigilance and real-world efficacy reassessment, ensuring the safety and effectiveness of oncology drugs throughout their life cycle. Collectively, these innovations mark the beginning of a new era of intelligent and precise cancer therapeutics.

Realizing the full potential of AI in oncology drug management requires close interdisciplinary collaboration. Oncologists, pharmaceutical scientists, and AI engineers must develop a shared language and cooperative frameworks. Clinicians, who understand unmet needs and patient realities, should be involved in model development and validation to ensure clinical relevance; data scientists must gain fluency in medical knowledge to design context-appropriate algorithms, while also addressing explainability and fairness. Industry–academia–regulator partnerships are crucial to transforming research prototypes into scalable clinical tools. Ethical scholars and policymakers also have an essential role in guiding innovation along compliant and socially responsible pathways.

Looking ahead, the integration of AI with emerging technologies will further expand the frontiers of oncology drug management. Organoid-based platforms are gaining traction—patient-derived tumor organoids can mimic *in vivo* tumor biology and drug responses. Applying AI to analyze large-scale organoid screening data could uncover clinically translatable therapeutics with unprecedented accuracy. Indeed, several companies have already used AI–organoid systems to identify novel compounds that are now advancing into clinical trials, validating the feasibility of this approach (93). At the same time, single-cell and spatial multi-omics technologies are enabling high-resolution dissection of tumor heterogeneity and microenvironments. AI will be indispensable for processing and interpreting these ultra-high-dimensional datasets, identifying resistance clones, immune escape mechanisms, and novel targets at the single-cell level. Moreover, advances in quantum computing and edge computing may soon unlock new capabilities in molecular simulation and real-time clinical decision-making.

In summary, AI is ushering in an intelligent era for oncology drug development and management, with broad and far-reaching applications. To fully harness its benefits, we must remain guided by clinical needs, scientific rigor, and ethical principles—leveraging AI’s strengths while proactively managing its risks. Only through such an integrated and responsible approach can AI truly become a transformative force in cancer medicine, improving survival and quality of life for patients worldwide. The future of oncology drug management—shaped by the synergy between human wisdom and AI—is one we can all look forward to.

Priorities: (1) Prospective, multi-site outcome trials to establish real-world effectiveness and safety; (2) Fairness auditing and algorithmovigilance (pre-specified update rules, temporal validation, subgroup monitoring); (3) Workflow/regulatory integration (EHR/registry interoperability; SaMD-aligned change control and post-market monitoring).
